# The effects of everyday-life exposure to polycyclic aromatic hydrocarbons on biological age indicators

**DOI:** 10.1186/s12940-020-00669-9

**Published:** 2020-12-03

**Authors:** Sofia Pavanello, Manuela Campisi, Giuseppe Mastrangelo, Mirjam Hoxha, Valentina Bollati

**Affiliations:** 1 Medicina del Lavoro, Dipartimento di Scienze Cardio- Toraco- Vascolari e Sanità Pubblica, Università di Padova, Padova, Italy; 2 Azienda Ospedaliera di Padova, Unità di Medicina del Lavoro, Padova, Italy; 3 EPIGET – Epidemiology, Epigenetics and Toxicology Lab, Dipartimento di Scienze Cliniche e di Comunità, Università degli Studi di Milano, Milan, Italy; 4 Dipartimento di Medicina Preventiva, Fondazione IRCCS Ca’ Granda Ospedale Maggiore Policlinico, Milano, Italy

**Keywords:** Polycyclic aromatic hydrocarbon, Biological aging, Telomere length, Mitochondrial DNA copy number, DNA adduct, Structural equation modelling

## Abstract

**Background:**

Further knowledge on modifiable aging risk factors is required to mitigate the increasing burden of age-related diseases in a rapidly growing global demographic of elderly individuals. We explored the effect of everyday exposure to polycyclic aromatic hydrocarbons (PAHs), which are fundamental constituents of air pollution, on cellular biological aging. This was determined via the analysis of leukocyte telomere length (LTL), mitochondrial DNA copy number (LmtDNAcn), and by the formation of anti-benzo[a]pyrene diolepoxide (B[a]PDE–DNA) adducts.

**Methods:**

The study population consisted of 585 individuals living in North-East Italy. PAH exposure (diet, indoor activities, outdoor activities, traffic, and residential exposure) and smoking behavior were assessed by questionnaire and anti-B[a]PDE–DNA by high-performance-liquid-chromatography. LTL, LmtDNAcn and genetic polymorphisms [glutathione S-transferase M1 and T1 (GSTM1; GSTT1)] were measured by polymerase chain reaction. Structural equation modelling analysis evaluated these complex relationships.

**Results:**

Anti-B[a]PDE–DNA enhanced with PAH exposure (*p* = 0.005) and active smoking (*p* = 0.0001), whereas decreased with detoxifying GSTM1 (*p* = 0.021) and in females (*p* = 0.0001). Subsequently, LTL and LmtDNAcn reduced with anti-B[a]PDE–DNA (*p* = 0.028 and *p* = 0.018), particularly in males (*p* = 0.006 and *p* = 0.0001). Only LTL shortened with age (*p* = 0.001) while elongated with active smoking (*p* = 0.0001). Besides this, the most significant determinants of PAH exposure that raised anti-B[a]PDE–DNA were indoor and diet (*p* = 0.0001), the least was outdoor (*p* = 0.003).

**Conclusion:**

New findings stemming from our study suggest that certain preventable everyday life exposures to PAHs reduce LTL and LmtDNAcn. In particular, the clear association with indoor activities, diet, and gender opens new perspectives for tailored preventive measures in age-related diseases.

**Capsule:**

Everyday life exposure to polycyclic aromatic hydrocarbons reduces leukocyte telomere length and mitochondrial DNA copy number through anti-B[a]PDE-DNA adduct formation.

**Supplementary Information:**

The online version contains supplementary material available at 10.1186/s12940-020-00669-9.

## Introduction

Continuous exposure to air pollution is one of the most important factors influencing adverse age-related outcomes, in particular cardiovascular (CVD) and respiratory diseases [1, 2]. Further understanding of other modifiable aging risk factors is needed to mitigate the increasing burden of age-related morbidities among a rapidly growing global demographic of elderly individuals [3]. In fact, the aging of the human population, referred to as the “gray” revolution, is a rising public health problem, even in the workplace.

Aging cannot merely refer to the effect of chronological time, given that it is a personal and multifaceted biological process [4]. Biological aging is assumed to mirror continuing modifications within a person, i.e. intrinsic physiological degeneration and the body’s capability to respond to different stressors (such as genetic factors and exposure to environmental and occupational agents) [5]. On the cellular level, aging is related to a range of molecular, biochemical and metabolic alterations. Growing indications have shown that nuclear DNA (nDNA) (e.g., telomere length attrition) and mitochondrial DNA copy number  (mtDNAcn) alterations (e.g., mtDNAcn reduction) are considered early hallmarks of biological aging, and may be the primary cause of cellular dysfunction [4]. Alterations of leukocyte telomere length (LTL) and LmtDNAcn are also implicated in age-related disorders, especially in CVD [6, 7]. An emerging body of evidence has associated environmental factors (e.g. exposure polycyclic aromatic hydrocarbons (PAHs)) to changes in LTL [8], whereas fewer studies have explored the impact of such pollutants on LmtDNAcn [9].

PAHs are widespread contaminants and major constituents of air pollution, as they are shaped throughout incomplete combustion of organic materials like tobacco, wood, fossil fuels, petroleum products, and even through the cooking of food [10]. Exposure to PAHs may pose a risk not only for lung cancer, but also for CVD, including atherosclerosis, hypertension, thrombosis and myocardial infarction [11]. Since PAH exposure is pervasive and modifiable, it is an appropriate target for age-related disorders prevention research studies. Benzo[a]pyrene (B[a]P), the key tracer of PAH mixtures, is the fundamental respiratory carcinogen of this complex mixture [12]. It has been suggested that B[a]P may both directly (by injuring DNA) and indirectly (by promoting the formation of oxidative damages and the onset of chronic inflammation) speed up the physiological aging process. This would accelerate the onset of chronic degenerative pathologies, CVD included [13]. B[a]P has been extensively described for its ability to harm nDNA when it forms the carcinogenic steady anti-B[a]P diolepoxide (anti-B[a]PDE)–DNA adduct at guanine exocyclic N2 [12]. Two additional molecular targets of B[a]P could be telomeres and mitochondrial DNA. Telomeres, as tandem triple-G-containing sequences repeated at each chromosomal end, keep the chromosome from eroding and fusing with neighboring chromosomes [14], and represent a susceptible domain for injury by the genotoxic B[a]PDE. Additionally, B[a]P may even affect mtDNA, an independent DNA molecule with a central role in controlling oxidative balance and apoptosis, both of which are also related to lung carcinogenesis [15]. B[a]P has been reported to possess greater damaging potential for mtDNA, with forty to ninety-fold higher affinity for mtDNA [15] than for nDNA. Furthermore, PAH (B[a]P) metabolic activation via aldo-keto reductase and/or manganese superoxide dismutase [16] produces reactive oxygen species (ROS) that can produce high levels of oxidized guanine in both nDNA [17] and mtDNA [18]. Furthermore, mitochondria, with fewer protective histones and lower DNA repair capability compared with nDNA, are extremely liable to be broken [15] and would be another relevant target in B[a]P exposure.

Existing evidence suggests a possible negative association between long-term exposure to fine inhalable particles, with diameters that are generally 2.5 μm and smaller (PM2.5) and LTL, and a positive association between short-term exposure and LTL [8]. However, for PAH exposure, the results are not conclusive. In our previous work we found a major impact of long-term exposure to high levels of PAHs in coke-oven workers on nDNA and LmtDNAcn, where exposure was evaluated by anti-B[a]PDE–DNA adducts and urinary 1-pyrenol, thus linking professional PAH exposure with cellular biological aging [19].

There is growing interest in structural equation modeling (SEM) as it represents a very important statistical tool for evaluating complex relations in several research areas [20]. In epidemiology, the applications of SEM have been limited thus far. The attractiveness of SEM stems mainly from the fact that researchers have recognized the necessity of grasping the complex interrelations between multiple variables under study. Traditional statistical approaches apply solely to a limited number of variables, and thus fail to deal with emerging sophisticated theories. SEM analysis is a statistical technique that links observed data with qualitative causative assumptions and tests whether variables are interdependent, and if so, the details of their interactions. This is achieved through an estimation procedure [20], which uses a set of concurrent regression equations to yield coefficient estimators more efficiently than single-equation estimators. This methodology is appropriate for the investigation of complex interrelationships, as it tests causative relationships instead of mere correlations [21]. The aim of the current study is to investigate the extent to which long-term exposure to PAHs, together with genetic polymorphisms and anti-B[a]PDE–DNA adducts, could affect LTL and LmtDNAcn within the general population. These multifaceted relationships are evaluated using the analysis of SEM.

## Methods

### Study design

The study population consisted of *n* = 585 participants listed within the LAINBIO project [22]. Enrollment was done at the Preventive Medicine Service of the University of Padua, Italy, from October 2002 to July 2005, as previously described [22]. All participants were notified of the purpose and strategies of the study and were requested to sign a consent form. The Ethics Panel of the School of Medicine, in accordance with principles of the Helsinki Declaration, approved the study (practice number 3843/AO/16). The admissibility criteria for participants were as follows: (1) older than eighteen years at registration, (2) not professionally exposed to PAHs, (3) inhabitant of the Veneto region at the time of the enrolment, and (4) willing to sign the consent form and provide blood and urine samples. Conditions for exclusion from the study included preceding diagnosis of cancer, cardiovascular disease, or stroke within the last year, as well as other chronic syndromes such as multiple sclerosis, Alzheimer’s disease, Parkinson’s disease, depression, bipolar disorder, schizophrenia, and epilepsy [22]. Information on possible extracurricular PAH exposure (i.e. diet and indoor and outdoor exposure) as well as intake of fruit and vegetables were gathered by means of a structured questionnaire, as previously described [22]. When subjects filled out the questionnaire, blood samples were drawn and conserved at − 80 °C until DNA was obtained by a Genomic DNA purification kit (Wizard, Promega, Italy), following the manufacturer’s instructions. DNA was used for subsequent analyses of leukocyte DNA adducts, LTL and LmtDNAcn. All participants became anonymous after sample collection.

### Estimation of PAH exposure from the questionnaire

Using a self-compiled questionnaire, we collected data on environmental exposure to PAH focusing on the following categories.

- Diet. This is the number of times per year that PAH-rich meals were consumed, including grilled meat or pizza roasted in wood-burning ovens. In the statistical analysis, this was considered as a continuous data variable.
- Indoor exposure was the combination of a number of sources: presence of a coal- or wood-heater in the residence (used less than or more than 5 times per year = a score of 1 or 2, respectively), leisure activity with exposure to PAHs (works at home/hobbies involving exposure to mineral oils, soot, fumes from combustion of wood, leaves or other combustible materials, engine exhaust = a score of 1), and exposure to passive tobacco smoke (a score of 1). Participants were categorized as having no exposure (total score of 0), or low (total score of 1), intermediate (total score of 2) or elevated (total score of 3) indoor exposure, the latter including one individual who had a score of 4. In the statistical analysis, this was taken into consideration as a continuous variable.
- Home. Residential exposure was classified as urban/ peripheral or country areas according to the residential address of each participant, which was used as a categorical data variable with two levels [urban/ peripheral (score = 1) or country areas (score = 0)] in the statistical analysis i.e. 1 or 0, respectively.
- Traffic. The exposure assessment of traffic-related air pollution nearby the zone of habitation was based on responses to the following questions: “How do you estimate the traffic in the area where your home is located?” Continuous heavy traffic for most of the day 2; Intense intermittent traffic (e.g. only during rush hour) 1; Scarce or no traffic 0. Traffic was accounted for as a categorical variable with two levels: score = 1 for continuous/intense and score = 0 for scarce or no traffic.
- Outdoor. Subjects with outdoor exposure to traffic pollution were individuals such as traffic police officers and gardeners. The variable was categorical in two levels: ≥4 h/day (score = 1), or <  4 h/day (score = 0).
- Smoking. Current smokers (including individuals who had quit smoking up to 4 weeks before participation to the study) were given a score as 1, while nonsmokers and former smokers were scored as 0.

### Analysis of the *anti*-B[*a*]PDE–DNA adduct

*Anti*-B[*a*]PDE–DNA adduct was identified by high-performance-liquid-chromatography (HPLC) along with a fluorescence detector [22]. The procedure was as previously described [23] with some minor changes, primarily concerning the mechanization of the HPLC assay. In this way the batch impact was abated (see complete description of anti-B[a]PDE–DNA adduct analysis in the Supplementary Material). In short, samples with non-measurable DNA adducts had a value of one-half the threshold of detection of the assay (LOD/2 = 0.125). Adduct levels were considered in the analyses both continuously and categorically (present or non-measurable). Individuals classified as having adducts present were those with a level of ≥0.5 adducts/10^8^ nucleotides.

### Leukocyte telomere length (LTL)

LTL was appraised by using quantitative Real-Time PCR (00qRT-PCR) as previously described [24]. This test calculates LT0L in genomic DNA by establishing the proportion of telomere replicate copy number (T) compared to copy number of a nuclear gene (S) in a specified sample relative to a reference DNA sample, i.e. the so called Telomere/Single gene (T/S) ratio. The single-copy gene was human (beta) globin (hbg). As reference DNA, we pooled DNA from 50 subjects randomly selected from the study population (500 ng for each sample). From this, a new standard curve ranging from 30 to 0.23 ng/μl (serial dilutions 1:2), was added in every “T” and “S” PCR run, versus a negative sample (water). In total 9 ng of DNA sample was incorporated in each analysis. Each sample was threefold analyzed as reported in Pavanello et al. [24]. LTL was treated in the analyses both as categorical tl50 (higher or lower than median: 0 = below 0.896; 1 = equal/above 0.896) or as a continuous variable. See complete description of LTL analysis in the Supplementary Materials and Methods.

### Leukocyte mtDNAcn (LmtDNAcn)

LmtDNAcn was determined in the same DNA of LTL testing by means of the qRT-PCR as previously described [9]. This assay appraises mtDNAcn in experimental samples by establishing the relation between the mitochondrial (MT) DNA copy number and the single copy number of a gene (S) relative to the MT/S ratio of a reference assembled DNA sample [9]. All samples were replicated threefold. The average of the three MT measurements was divided by the average of the three S measurements to calculate the MT/S ratio for each sample. The coefficient variation for the MT/S in samples examined on two distinct days was 6%. LmtDNAcn was treated as a continuous data variable in the statistical analysis. See complete description of LmtDNAcn analysis in the Supplementary Materials and Methods.

### GSTM1 and GSTT1

A multi-PCR technique was applied to detect the presence or absence of the GSTM1 and GSTT1 genes, following the procedure as previously described [23]. Briefly, the same amplification mix contained both GSTM1- and GSTT1-specific primer pairs and incorporated a third primer pair for β-globin, the internal positive PCR control. The GSTT1 (480 bp), β-globin (285 bp), and GSTM1 (215 bp) amplified products were separated in a 2% agarose gel. The absence of the GSTM1- or GSTT1-specific fragment designated the corresponding null genotype (*0/*0), whereas the β-globin-specific fragment indicated the presence of amplified fragments in the reaction blend.

### Statistical analysis

We used the Spearman’s rank coefficient to calculate the pairwise correlation among the five variables of environmental exposure to PAHs (diet, indoor, home, traffic, outdoor), as well as age and sex. Through a mathematical model (see below: SEM), these variables were aggregated in the latent variable “PAH” that represents an overall picture underlying physical reality, making it easier to understand and handle the data.

#### Analytic strategy

**Fig. 1:**
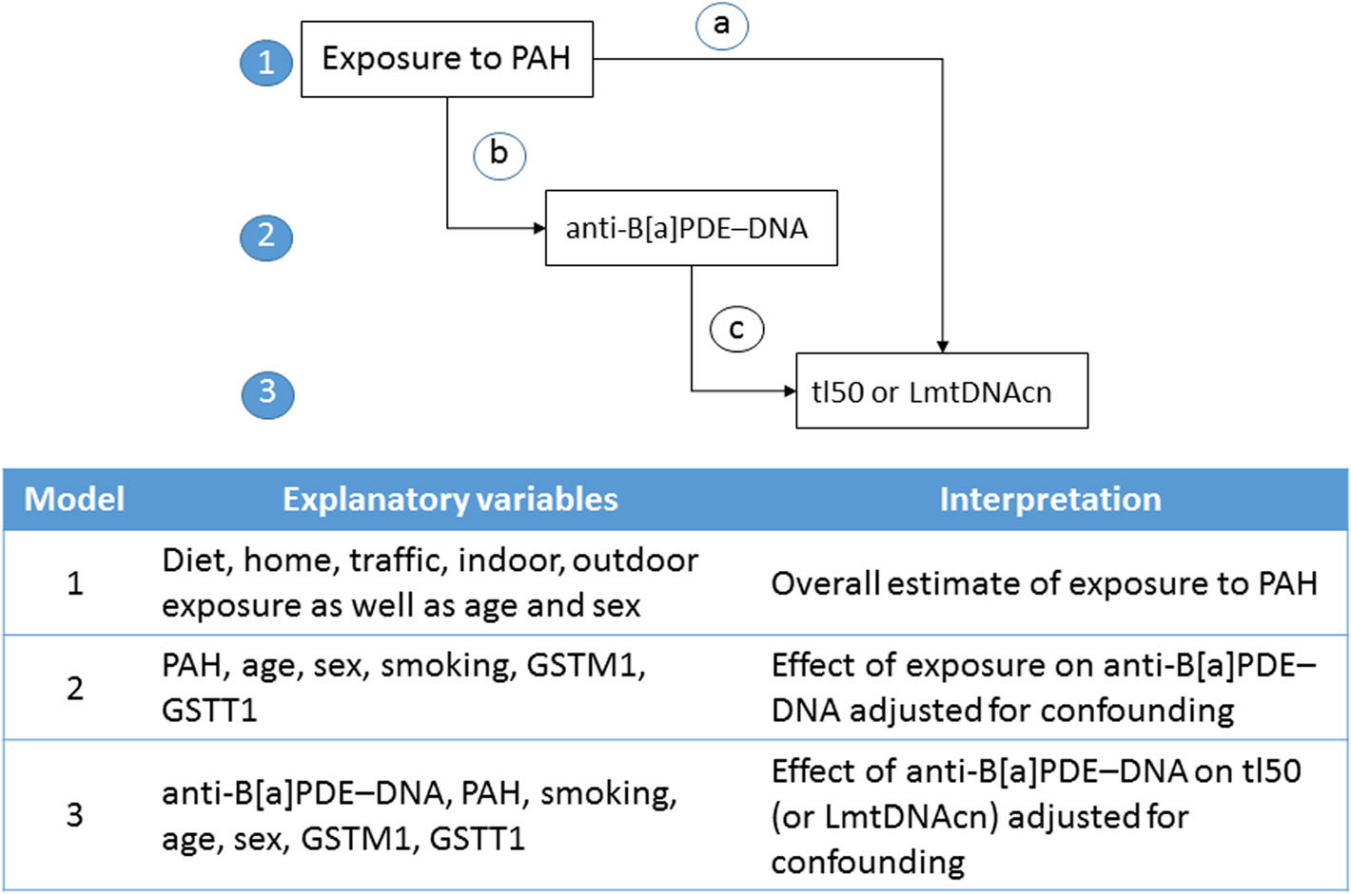
Conceptual hierarchical framework and summary of steps in the analysis. The latent variable “exposure to PAH” was considered as the distal determinant **a**), acting through the proximate determinant **b**) “anti-B[a]PDE–DNA” (intermediate variable or mechanism) to affect the final outcomes c) “tl50” (LTL median) or, alternatively, LmtDNAcn

We used a conceptual framework describing the hierarchical relationships between risk factors, based on knowledge of the relevant literature and temporal considerations. As shown in Fig. 1, the latent variable “exposure to PAH” derived from the self-compiled questionnaire was considered as the distal determinant, acting through the proximate determinant “anti-B[a]PDE–DNA” (intermediate variable or mechanism) to affect the final outcomes “tl50” (LTL median) or, alternatively, LmtDNAcn. Although it is uncommon, the notion of proximate and distal determinants is important because in an approach based entirely on statistical associations, distal factors are often improperly adjusted for proximate factors with a consequent reduction or elimination of the effects of the former [20].

All the above assumptions were converted into two models of SEM, one for each final outcome (either tl50 or LmtDNAcn). The STATA command syntax for each model was:

1. SEM (PAH - > diet indoor outdoor home traffic) (anti-B[a]PDE–DNA < - PAH sex smoking gstm1) (tl50 < − anti-B[a]PDE–DNA age sex smoking), stand vce(oim);
2. SEM (PAH - > diet indoor home traffic outdoor) (anti-B[a]PDE–DNA < - PAH sex smoking gstm1) (LmtDNAcn <− anti-B[a]PDE–DNA sex), stand vce(oim)

The SEM model commands assume that variables are latent if the first letter of the name is capitalized. The variable “PAH” is capitalized because is our latent variable name. In the first, second and third set of parentheses we specified, respectively, the estimations of the latent variable “PAH”, the model for the mediator variable “anti-B[a]PDE–DNA” and the model for the final outcome (either tl50 or LmtDNAcn). Notice that “anti-B[a]PDE–DNA” was a dependent variable in the second set and an explanatory variable in the third set of parentheses. Furthermore, the correlation plot between the anti-B[a]PDE–DNA tetrol biomarker and the self-reported PAH proxy was obtained using appropriate STATA commands. We generated the numerical values of the latent variable “PAH” in the context of two SEM models: PAH1 for the outcome tl50 and PAH2 for the outcome LmtDNAcn. The individual values of both PAH1 and PAH2 were plotted against the logarithm of tetrol (anti-B[a]PDE–DNA). The latent variable PAH is estimated by SEM program, not observed. As can be read in statistical package STATA 14 for SEM analysis, “a variable is latent if it is not in your dataset but you wish it were. You wish you had a variable recording the propensity to commit violent crime, or socioeconomic status, or happiness, or true ability, or even income. Sometimes, latent variables are imagined variants of real variables, variables that are somehow better, such as being measured without error. At the other end of the spectrum are latent variables that are not even conceptually measurable”. All the predictors shown in Fig. 1 were used in preliminary analyses (data not shown) but only the statistically significant terms were included in SEM final models. In STATA commands, “stand” specifies that the effects are expressed as standardized (or beta) coefficients that make comparisons easily by ignoring the independent variable’s scale of units, while “vce(oim)” specifies how the standard errors are calculated. “VCE” stands for variance–covariance matrix of the estimators, and “oim” stands for observed information matrix (OIM). The OIM estimator of the VCE is the default and is based on asymptotic maximum-likelihood theory. The VCE obtained in this way is valid if the errors are independent and identically distributed normal, although the estimated VCE is known to be reasonably robust to violations of the normality assumption.

We used three SEM goodness-of-fit statistics: (1) the chi square test for “model versus saturated” (the saturated model is the model that fits the covariances perfectly), (2) the standardized root mean squared residual (SRMR), and (3) the coefficient of determination (CD).

SEM results were both tabulated and presented graphically.

The sample size required for SEM is dependent on model complexity, the estimation method used, and the distributional characteristics of observed variables. The best option is to consider the model complexity (i.e., the number of exogenous variables) and the following rules of thumb: minimum ratio 5:1, with a recommended ratio of 10:1, or a recommended ratio of 15:1 for data with no normal distribution [25]. With ten exogenous variables used in the SEM model, we should have a minimum of 150 (= 15 × 10) subjects; in total we reached 585 (537 with complete data), thus fulfilling these requirements. The analysis was conducted with the statistical package STATA 14.

## Results

In the present study, complete individual data are available for 537 (92%) out of 585 original subjects. Table 1 shows the main characteristics of subjects with number and row percent of categorical variables or mean and standard deviation of continuous variables. Age was relatively young given that the sample group targeted working-age individuals, sex was well balanced, and the majority of study subjects were nonsmokers and former smokers (79.7%). The intake of PAH-containing meals was on average equal to 46.8 times/year, which equates to less than once a week. Urban residence was predominant; outdoor exposure to traffic came mainly from occupations such as traffic police officers, gardeners and others. GSTM1 and T1 null frequencies were in line with what has been found in larger Caucasian population studies [26]. Hardy-Weinberg Equilibrium (HWE) was tested for each polymorphism; the allele frequency was calculated, and the observed genotype frequency was compared with expected frequency using a X^2^ test. The allele distributions for the polymorphisms were under HWE with *p*-value > 0.05 (data not shown). For the outcome variables, the main concern was the ascertainment of distribution. A value of 0.125 adducts/10^8^ nucleotides was assigned to 299 participants (56%) with non-detectable adducts. Anti-B[a]PDE–DNA did not follow a normal distribution, and while any transformations failed to reduce its skewness (data not shown) the variable was used as such. Considering this skewed distribution, LTL was used as a 0/1 variable (below or above the median) since the median (0.9 T/S) and the mean (1.00 T/S) were rather close values. After square root transformation, the distribution of LmtDNAcn approached a normal distribution but still displayed a significant departure from the normal distribution (data not shown). Therefore, LmtDNAcn was used as it was, given that the estimated VCE is reasonably robust in SEM, when considering violations of the normality assumption.

**Table 1:** Main characteristics of subjects: demographic variables (age, sex); putative risk factors (smoking, diet, indoor, home, traffic, outdoor); genetic traits with modulating role (GSTM1, GSTT1), mediation variable (anti-B[a]PDE-DNA); outcomes (LTL and LmtDNAcn)

FACTORS	STRATA	Number (row %)	Mean ± St Dev
Age (years)			41.90 ± 9.03
Sex	0 = Females	285 (52.6)	
	1 = Males	257 (47.4)	
Smoking ^a^	0 = Non- Ex-smokers	432 (79.7)	
	1 = Current smokers	110 (20.3)	
Diet (times/year) ^b^			46.8 ± 43.1
Indoor ^c^	0 = not exposed	325 (60.2)	0.63 ± 0.87
	1 = low	104 (19.4)	
	2 = medium	96 (17.9)	
	3 = high	14 (2.61)	
Home	0 = Rural	154 (28.4)	
	1 = Urban	388 (71.6)	
Traffic	0 = Scarce / moderate	277 (51.1)	
	1 = Intense	265 (48.9)	
Outdoor ^d^	0 = < 4 h/day	443 (81.7)	
	1 = ≥4 h/day	99 (18.3)	
GSTM1^e^	0 = (*0/*0)	296 (55.1)	
	1 = *1/*1 and *0/*1	241 (44.9)	
GSTT1	0 = (*0/*0)	97 (18.1)	
	1 = *1/*1 and *0/*1	440 (81.9)	
Anti-B[a]PDE-DNA ^f^			1.35 ± 2.87
LTL	0 = below 0.896	271 (49.9)	1.00 ± 0.45
	1 = equal /above 0.896	272 (50.1)	
LmtDNAcn			1.13 ± 0.31

^a^Smokers: current cigarette smokers were subjects smoking for at least 1 month before enrolment in the study

^b^Subjects consuming charcoaled meat or pizza (times/year)

^c^ Sum of several factors: presence of fireplace or coal- or wood-stoves at home; hobbies with introduction of PAHs; exposure to passive smoking

^d^ Exposure to outdoor pollution

^e^ The absence of the specific GSTM1 fragment indicated the corresponding null genotype (*0/*0), and its presence corresponded to the *1/*1 and *0/*l genotypes

^f^ A value of 0.125 adducts/10^8^ nucleotides was assigned to 299 participants (56%) with non-detectable adducts. Samples with ≥0.5 adduct/10^8^ nucleotides were positive

Spearman’s rank coefficients and significance level for pairwise correlation of environmental exposure to PAHs as well as age and sex are reported in the Supplemental Information (Table 1S). The variable “outdoor” was highly related to sex – since only males were present in this category – but not correlated with other aspects of PAH environmental exposure. Perhaps further study can clarify this value for females as well. Diet and indoor exposure were significantly correlated with each other, and both were negatively correlated with “home” and “traffic” (Table 1S). It is worth noting that most rural households ranked 2 or 3 in the classification of indoor exposure to PAH, and were not affected by intense traffic in the neighborhood (data not shown). Interestingly, the times per year that subjects consumed PAH-containing meals (i.e. the “diet” category) significantly decreased with increasing age and was higher in males than in females (Table 1S).

Four groups of SEM results concerned with the analysis of tl50, which is the dichotomous variable indicating LTL, are reported in the Supplemental Information (Table 2S). These are:

1. Structural equations. This includes the beta coefficients (with a “minus” sign indicating an inverse relationship), 95% confidence intervals and *p*-values for each of two structural equation models.

- The first model shows that the endogenous variable anti-B[a]PDE–DNA significantly increased with increasing value of the latent variable PAH (beta = 0.178; *p* = 0.005) and with active smoking (0.149; *p* = 0.0001), whereas the presence of the detoxifying GSTM1 (beta = − 0.098; *p* = 0.021) decreased adduct levels, as did sex (males) (to a lesser extent).
- The second model shows that the endogenous variable LTL_50 percent_ (tl50) significantly decreased with anti-B[a]PDE–DNA (beta = − 0.092; *p* = 0.028), age (beta = − 0.135; *p* = 0.001), and to be males (beta = − 0.117; *p* = 0.006), while active smoking (beta = 0.187; p = 0.0001) showed an opposite effect. Interestingly, PAH was not a significant predictor in the second structural equation.

2. Measurement. The standardized (beta) coefficients for this measurement model can be interpreted as correlation coefficients describing the direction (positive or negative) and degree (strength) of relationship between each indicator and the latent variable PAH. The most significant indicator was “diet” and “indoor”, and the least significant was “outdoor”. The positive coefficients (such as for “diet”, “indoor” and “outdoor”) indicate that the latent variable PAH tends to increase with increasing values of these exposure variables. The negative coefficient of “home” and “traffic” means that these factors tend to go in the opposite direction, probably because the latter variables were false indicators of exposure.
3. Errors. The variability explained (1 – error) by the above fitting was about 7% for both anti-B[a]PDE–DNA (ε6 = 0.931) and tl50 (ε7 = 0.932).
4. Covariances. The findings demonstrated that age and sex were individually correlated with PAH.

The value of Chi square test for the discrepancy of the specified model versus the saturated model was 139.4 with *p*-value < 0.0001. The size of residuals (SRMR) were equal to 0.055 and the coefficient of determination (CD) of 0.524, both of which demonstrated a good fit.

**Fig. 2:**
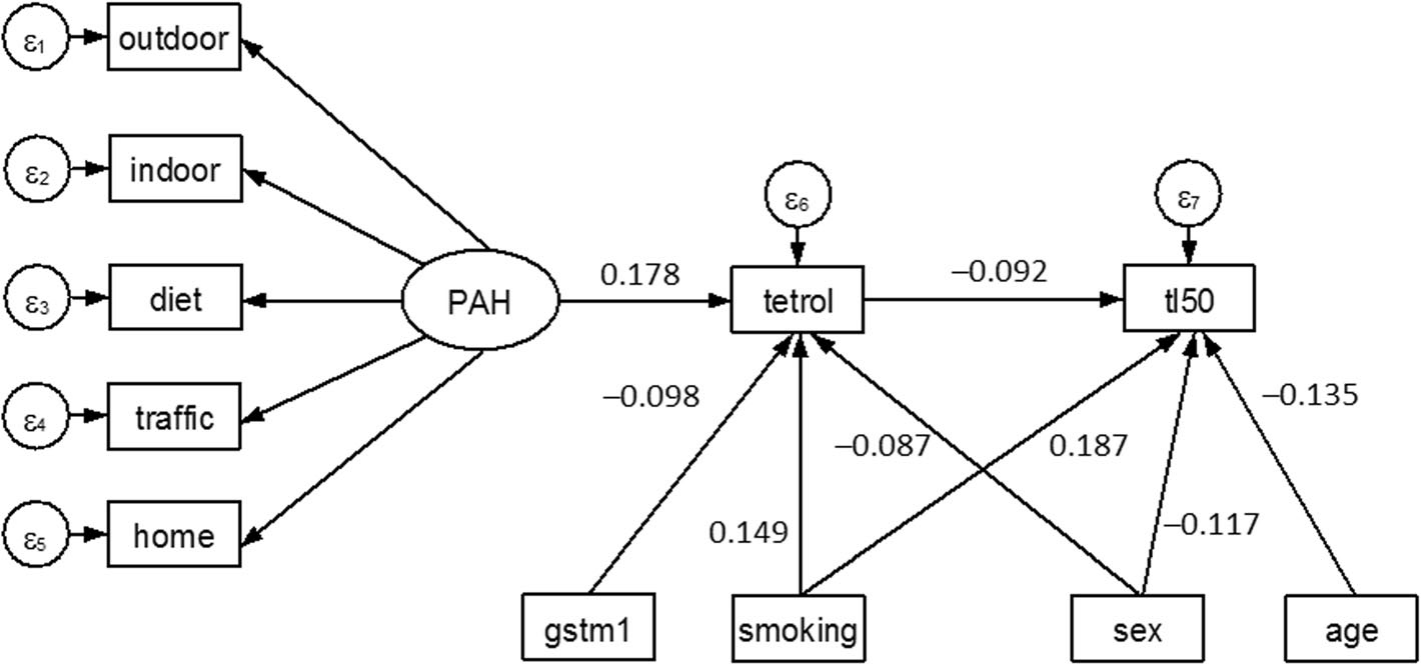
Path diagram of results shown in Table 2S. An oval indicates the latent variable, square boxes indicate the observed variables, circles indicate errors, arrows specify the direction of causal flow, an arrowed route is a path, and the estimated beta coefficients appeared along the paths. The effect of one variable on another is called direct. There was evidence of indirect effects (one variable affecting another variable which in turn affects a third), indicating that PAH exposure decreases LTL through formation of anti-B[a]PDE–DNA (tetrol).

Using the graphical interface of SEM, the results shown in Table 2S were displayed as a path diagram in Fig. 2.

**Fig. 3:**
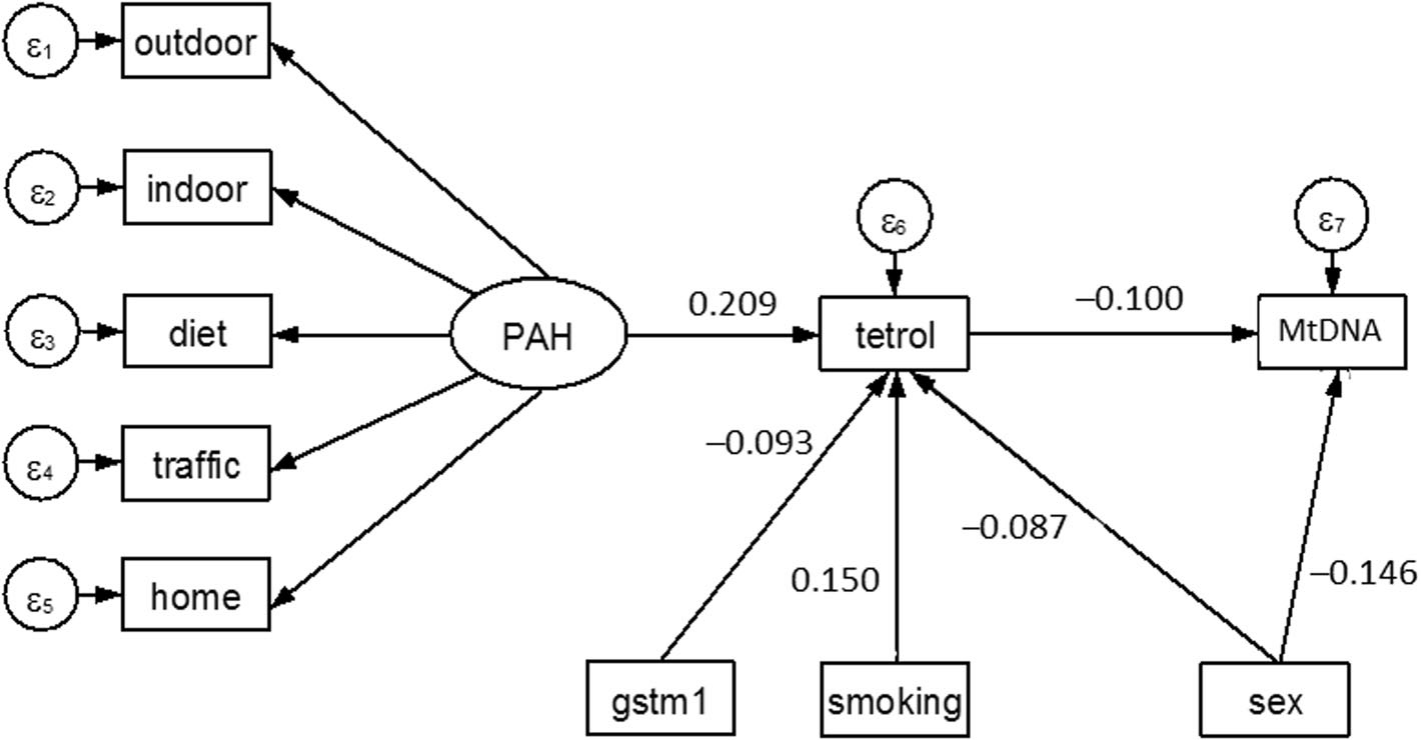
Path diagram of results shown in Table 3S. An oval indicates the latent variable, square boxes indicate the observed variables, circles indicate errors, arrows specify the direction of causal flow, an arrowed route is a path, and the estimated beta coefficients appeared along the paths. As for LTL, PAH exposure appeared to have no direct effect on LmtDNAcn but an indirect effect through the mediation of anti-B[a]PDE–DNA (tetrol).

The analysis of LmtDNAcn was carried out with a STATA command similar to that used for tl50, except than the second structural equation with LmtDNAcn as outcome is reported in the Supplemental Information (Table 3S). The significant predictors of LmtDNAcn were anti-B[a]PDE–DNA (beta = − 0.100, *p* = 0.018) and sex (to be males) (beta = − 0.146, *p* = 0.0001). As for LTL, PAH exposure appeared to have no direct effect on LmtDNAcn but an indirect effect through the mediation of anti-B[a]PDE–DNA. Using the graphical interface of SEM, the results shown in Table 3S were displayed as a path diagram in Fig. 3. Despite the fact that the variance explained by the above fitting was as low as 3.1% (1–0.969), the fit was good for the whole SEM model (chi square test = 116.2, *p* < 0.0001; SRMR = 0.055; CD = 0.529).

No significant association was found between LTL and LmtDNAcn (number of obs = 543; Spearman’s rho = 0.0120; *p* = 0.7810).

**Fig. 4:**
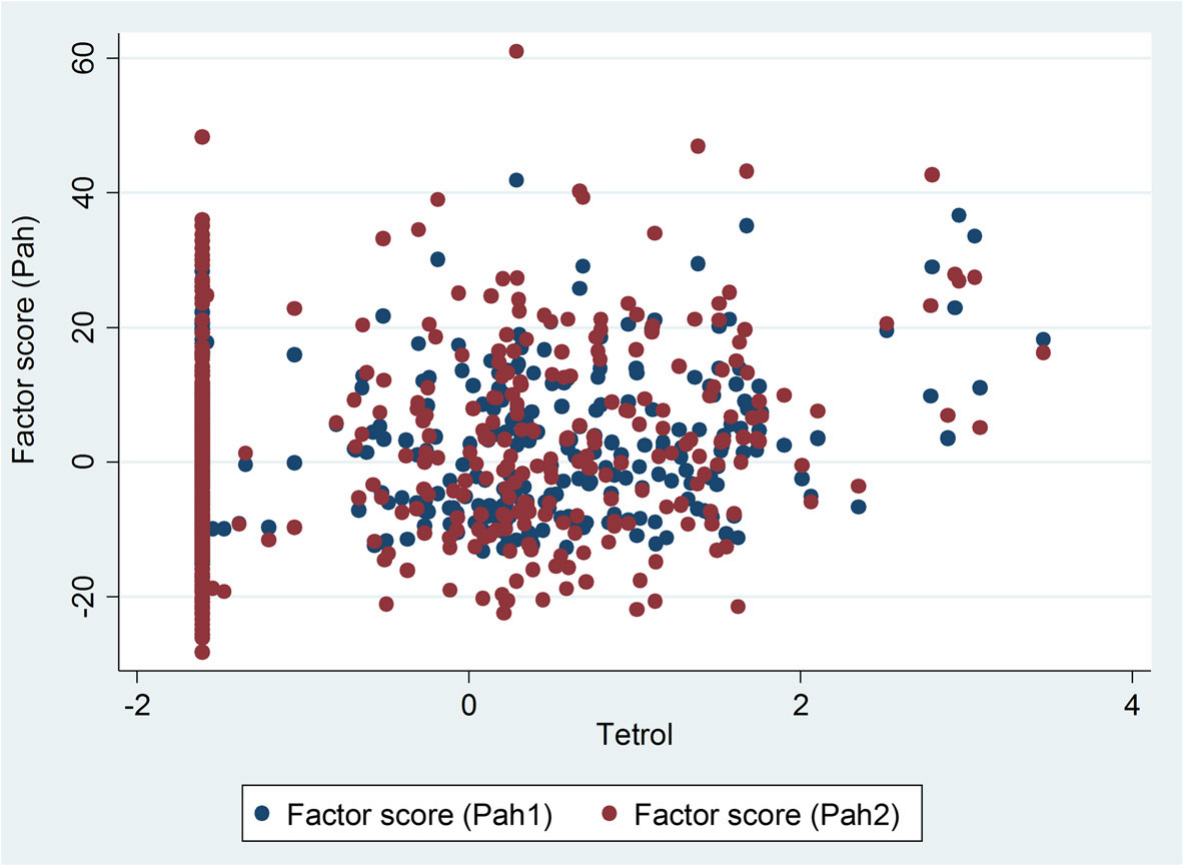
The correlation plot between the anti-B[a]PDE–DNA (tetrol) and the self-reported PAH proxy

Lastly, Fig. 4 shows the scatter plot of PAH1 and PAH2 against the logarithm of tetrol. It can be seen that the values of PAH1 and PAH2 largely overlap, showing a positive association with tetrol. The graph suggests that PAH adducts to DNA (anti-B[a]PDE–DNA) could depend at least in part by PAH exposure proxies collected on questionnaires. The points aligned vertically on the left of the graph are mainly participants with assigned DNA-adduct value (at LOD/2).

## Discussion

The key finding of our study is that in a postulated chain of events, a hierarchical relationship was outlined between different PAH exposures (diet, indoor, outdoor, traffic, residential) as well as smoking that, acting through anti-B[a]PDE–DNA adduct formation, affected LTL and LmtDNAcn. This opens up the possibility that genotoxic carcinogen PAHs may also be gerontogenic for the general population (in particular males), by speeding up biological indicators of aging. In fact, LTL and LmtDNAcn reduced with anti-B[a]PDE–DNA formation, particularly in males. In accordance with our previous findings, we found that anti-B[a]PDE–DNA was significantly increased with PAH exposure and active smoking, whereas the presence of the detoxifying GSTM1 decreased adduct levels. Lastly, male gender was also associated with decreased anti-B[a]PDE-DNA levels, to a lesser extent.

The direct negative relationship between LTL and DNA adducts is in line with our study in coke oven workers, who are highly exposed to PAH-related work [19], and thus would suggest that adduct formation might have a direct role in LTL erosion. In fact, anti-B[a]PDE–DNA adducts verified by means of HPLC-Fluorescence, are the consequences of the stereo-selective link between B[a]P and the exocyclic N2 of guanine, which is considered to be the crucial step in B[a]P carcinogenicity [9]. Furthermore, telomeres (as triple-G-holding chains) are a susceptible point for damage by such B[a]PDE genotoxic metabolites. In fact, double-strand breakdowns and interference with the replication fork, which occur with extremely damaged telomeric bases, might directly generate telomere attrition [27]. Furthermore, anti-B[a]PDE–DNA creation and subsequent telomere erosion may be regulated by a reduction in B[a]P detoxification, attributable to the presence of a specific GSTM1 detoxifying polymorphism. The formation of adducts within the proteins of the telomere-protecting complexes (including those regulated by GSTM1), can also be thought of as an extra outcome accounting for shorter LTL. In fact, altered mRNA expression levels of end-binding proteins associated with telomere injury were recently found in individuals with PAH exposure [28].

We also found that LmtDNAcn significantly reduced with anti-B[a]PDE–DNA. As for LTL, PAH exposure appeared to have no direct effect on decreased LmtDNAcn but an indirect effect through the mediation of anti-B[a]PDE–DNA. Our results agree with those by Pieters et al. [29] who found a diminution of LmtDNAcn associated with low chronic exposure to PAHs in house dust during wintertime. The ability of B[a]P to reduce mtDNA content was also validated in vitro in human TK6 cells [30]. In view of the genotoxic capability of B[a]P, this carcinogen may also have an impact on mtDNA dynamics. The lipophilic nature of B[a]P and its metabolite anti-B[a]PDE, combined with the very high ratio of lipid/DNA in mitochondria, expedites the rate at which these molecules enter mitochondria. Moreover, anti-B[a]PDE possesses 40 to 90-fold higher affinity for mtDNA than for nDNA [15]. Compared with nDNA, mtDNA has fewer protective histones and DNA repair ability, and is therefore extremely vulnerable to DNA injury. Consequently, the role of mtDNA-bound anti-BPDE could be a valuable fraction of the total cellular cargo of DNA adducts. Our results are consistent with a study that reported a significant reduction in LmtDNAcn in the lungs of long-term heavy cigarette smokers. Conversely, our previous study in non-smoking male workers with high exposure to PAHs (> 3 μmol 1-pyrenol/mol creatinine) detected significantly higher LmtDNAcn in these subjects, compared with controls [9]. It has been proposed that the oxidative stress, triggered by exposure to PAHs, has a dual impact on mitochondrial DNA content. Indeed, mild stress can promote mitochondrial DNA production and increased mitochondria number in order to sustain the higher respiratory needs of the cell and, as such, maintain cell viability [31]. However, excessive oxidative stress spawned by tobacco smoke might instead lead to reduced (or no) synthesis of mtDNA, as tobacco smoke contains many toxic, carcinogenic and mutagenic compounds, as well as stable and unstable free radicals and reactive oxygen species (ROS), with the potential for oxidative DNA damage. Such damage may then eventually lead to cell senescence or cell death.

In this study, smoking was found to be directly related to the increase in LTL. Several studies, including large surveys such as those conducted by Bischoff et al. [32] and Cassidy et al. [33] were unable to substantiate the negative association between LTL and smoking discovered by others [34]. These inconsistent results seem to detect a moderate effect of smoking on LTL, if any, which may simply not be measurable. In-vitro experiments have shown that telomere length increases in younger inflammatory T cells [35] over the course of inflammation, which is a key process in mediating smoking-related health effects [36]. Therefore, the higher LTL in smokers that we discovered in the current study could be ascribed to the recall of younger inflammatory cells from the bone marrow to the blood circulation, in response to inflammatory signals [35]. Likewise, short-term exposure to PM was associated with a quick rise in blood LTL [36, 37]. Taken together, these observations indicate that short-term smoking exposure may generate a rapid increase in LTL, which may then contribute to sustaining the inflammatory mechanisms related to adverse health effects.

Next to active smoking, the most significant determinants of PAH exposure leading to increased anti-B[a]PDE–DNA, were “diet” and “indoor” exposure, while the least significant was “outdoor” exposure. Interestingly, much attention has been paid to the detrimental effects of outdoor pollution, even though the majority of individuals spend most of their time inside, with indoor air that could be even more contaminated than outdoor air. Indoor air pollution was actually listed as one of the ten leading risk factors for the worldwide burden of disease [38]. Solid fuel use can increase risks for many cardiopulmonary diseases. Cooking and heating fumes was clearly associated with higher risks of CVD [39]. Similarly, Delgado et al. [40] showed that exposure to wood smoke (without radon, which was the confounding factor in the Chinese studies) is a risk for lung cancer in non-smokers. Our findings show that indoor pollution (mainly due to wood burning) could be a considerable font of B[a]P intake via inhalation that, by anti-B[a]PDE–DNA adduct formation, affects LTL and LmtDNAcn and thus should be thought of as a potential risk factor for cardiopulmonary diseases. Conversely, less attention has been given to risk analysis of gastrointestinal exposure to PAHs route. One study has shown that human exposure to environmental carcinogens such as PAHs occurs primarily from dietary sources [41], and epidemiological studies have revealed that a large proportion of human malignances is attributable, at least in part, to nutritional factors [42]. Furthermore, a clear relationship between aromatic-DNA adducts and food (e.g., grilled hamburgers) was found in subjects with low professional exposure to PAH, where chargrilled meat intake was verified by a questionnaire [43]. In two planned eating studies (involving grilled meat) with a small number of participants (*n* = 4 and 21), a significant enhancement of DNA adduct level was reported in a few subjects [44, 45]. These results, together with ours, would imply that PAH intake by means of diet is another crucial source of genotoxic exposure to consider, and is detectable by DNA adduct dosimetry in blood leukocytes.

Most biomonitoring studies of low environmental exposure to PAHs have analysed aromatic DNA adducts using 32P-postlabelling technology but high inter-laboratory variability and uncertainty in adduct identification have become evident [46]. Conversely, few studies have been published dealing with quantification of the specific adduct of B[a]P (anti-B[a]PDE–DNA, formed by the ultimate carcinogenic metabolite of B[a]P), with the analytical and rapid HPLC fluorescence method, in large groups of people environmentally exposed to PAHs. Our present study, on a large cohort, shows that anti-B[a]PDE–DNA adduct levels, detected by HPLC/fluorescence, are comparable with those of the few other studies – with low numbers of smokers – using the same technique [9, 19, 47–49]. In particular, in our previous work, in non-smoking coke-oven workers exposed to very high levels of PAHs and matched controls with 1-pyrenol excretion (30 times higher in workers than controls) all were (100%) positive for adduct compared to the 36% of controls. This indicates that anti-B[a]PDE–DNA adduct measurement in the general population is a sensitive biomarker of PAH exposure.

Interestingly, both LTL and LmtDNAcn adduct-related decrease are evident in males, while females had much less. The majority of studies examining differences in LTL between women and men found that women have longer telomeres than men [50]. Several plausible biological arguments can be formulated to explain this. These include the action of estrogens [51], which can stimulate the production of telomerase [52] and protect against reactive oxygen species damage [53]. Furthermore, the rate of LTL shortening was slower in women than men [50], as detected in cross-sectional and longitudinal studies [54]. The same mechanism may also result in lower susceptibility to genotoxic damage in women, via anti-B[a]PDE-DNA adducts in the telomeric region. This is in agreement with our observation on LmtDNAcn in a study on 1088 subjects of European descent, in which higher LmtDNAcn content was detected in women compared with men [55, 56]. Our findings are also consistent with previous studies that pointed to sex-specific differences in the effect of air pollution on cord blood LmtDNAcn, with boys being the most highly affected [29, 57]. On the other hand, some published reports have failed to demonstrate differences in LmtDNAcn content between men and women [58]. Taken together, our data therefore supports the concept that males could be more vulnerable to the effect of PAH exposure on LTL and LmtDNAcn.

No correlation between LTL and LmtDNAcn is revealed. DNA alterations in two cellular organelles, nucleus and mitochondria, that are driving force in age-related disorders, have been rarely investigated contemporarily. In our previous study we found a link between nuclear telomere attrition and mitochondrial alterations with p53 activation, among workers occupationally exposed to very high levels of carcinogenic PAH(B[a]P), but not in the matched controls [9]. This observation was in line with previous findings by Sahin [59] that showed a potential unifying mechanism connecting the nucleus and mitochondria in cellular aging, mediated by the activation of a p53-dependent pathway. According to our previous findings the lack of a correlation between LTL and LmtDNAcn found in the present work could therefore be ascribed to the low PAH exposure detected in the general population.

We acknowledge that the self-reported PAH exposure are not solely reflecting PAHs, and may also contain other constituents that are associated with LTL and LmtDNAcn decreases. However, the fact of noting that the reduction of LTL and mtDNAcn is directly related to the most relevant and specific adduct, that certifies the exposure to B[a]P, the leading PAH compound, reinforces the fact that the exposure to PAHs is the determinant for the decrease in LTL and LmtDNAcn.

In our opinion, statistical analysis with SEM is a strength of the present research. In medicine and natural sciences, a given outcome is often affected or influenced by more than one factor simultaneously. Multivariate techniques try to statistically account for these differences, adjusting an outcome measure Y to a 1-unit change in X, holding all other variables constant. However, it may be that other variables are not likely to remain constant; a change in X can produce a change in Z (direct effect) which in turn produces a change in Y (indirect effect). Both the direct and indirect effects of X on Y must be considered if we want to know what effect a change in X will have on Y. This can be done mathematically and statistically only using SEM. The procedure decomposes a correlation between two variables into their component parts: direct effects, indirect effects, common causes (X affects both Y and Z; this is a spurious association) and correlated causes (X is a cause of Z and X is correlated with Y). The user is required to state, often using a path diagram, the way that they believe the variables are inter-related. Via complex internal rules, SEM decides which model fits the data better. This method is more suitable for the analysis of complex interrelationships because it tests causal relationships rather than mere correlations.

## Conclusions

This study supports evidence that LTL and LmtDNAcn decrease are critical events that capture the everyday life exposure to PAHs within the general population. LTL and LmtDNAcn reduction, which are considered hallmarks of cellular aging, are associated with an increase in mortality rate and different age-related diseases, in particular CVD. Therefore, our results suggest that male subjects with high exposure would be more likely to face premature aging and have an earlier onset of age-related diseases. The finding that LTL and LmtDNAcn reduction depends on some preventable everyday life exposure (which can in turn be affected by certain genetic factors) opens new perspectives in the prevention of age-related disorders, especially CVD.

## Supplementary Information

**Additional file 1: Table 1S.** Spearman’s rank coefficients and significance level for pairwise correlation of the different environmental exposures to PAHs (see Methods for definitions) as well as age and sex. **Table 2S.** Four groups of SEM results (structural equations, measurement, variances and covariances) for the analysis of tl50; standardized beta coefficients (with “minus” sign indicating inverse relationship) with lower and upper limit of 95% confidence intervals (95%CI) and *p*-values. SEM’s goodness-of-fit statistics at bottom of table. **Table 3S.** Four groups of SEM results (structural equations, measurement, variances and covariances) for analysis of LmtDNAcn: standardized beta coefficients (with “minus” sign indicating inverse relationship) with lower and upper limit of 95% confidence intervals (95%CI) and p-values. SEM’s goodness-of-fit statistics at bottom of table.

## Data Availability

Data will be available if required.
