# Supplementary material for: The effects of everyday-life exposure to polycyclic aromatic hydrocarbons on biological age indicators
Source: Environ Health. 2020 Dec 3;19:128. doi: 10.1186/s12940-020-00669-9 (PMC7713168; doi:10.1186/s12940-020-00669-9)
Supplement: Supplementary file 1 — Additional file 1: Table 1S. Spearman’s rank coefficients and significance level for pairwise correlation of the different environmental exposures to PAHs (see Methods for definitions) as well as age and sex. Table 2S. Four groups of SEM results (structural equations, measurement, variances and covariances) for the analysis of tl50; standardized beta coefficients (with “minus” sign indicating inverse relationship) with lower and upper limit of 95% confidence intervals (95%CI) and p-values. SEM’s goodness-of-fit statistics at bottom of table. Table 3S. Four groups of SEM results (structural equations, measurement, variances and covariances) for analysis of LmtDNAcn: standardized beta coefficients (with “minus” sign indicating inverse relationship) with lower and upper limit of 95% confidence intervals (95%CI) and p-values. SEM’s goodness-of-fit statistics at bottom of table. [file 12940_2020_669_MOESM1_ESM.docx]

**THE EFFECTS OF EVERYDAY-LIFE EXPOSURE TO POLYCYCLIC AROMATIC HYDROCARBONS ON BIOLOGICAL AGE INDICATORS**

Sofia Pavanello^1,2*^, Manuela Campisi^1^, Giuseppe Mastrangelo^1^, Mirjam Hoxha^3^, Valentina Bollati^3,4^.

^1^ Medicina del Lavoro, Dipartimento di Scienze Cardio- Toraco- Vascolari e Sanità Pubblica, Università di Padova, Italia.

^2^ Azienda Ospedaliera di Padova, Unità di Medicina del Lavoro, Padova, Italia

^3^ EPIGET – Epidemiology, Epigenetics and Toxicology Lab, Dipartimento di Scienze Cliniche e di Comunità, Università degli Studi di Milano, Italia.

^4^ Dipartimento di Medicina Preventiva, Fondazione IRCCS Ca’ Granda Ospedale Maggiore Policlinico, Milano, Italia.

* Correspondence: [sofia.pavanello@unipd.it](mailto:sofia.pavanello@unipd.it); Phone : +39-049-821-6600; Fax: +39-049-821-2542

PAH everyday exposure by B[a]PDE-DNA alters LTL and mtDNAcn.

# Supplementary mATERIALS AND Methods

## HPLC/fluorescence analysis of *anti*-B[*a*]PDE–DNA adduct

*Anti*-B[*a*]PDE–DNA adducts was detected by HPLC/fluorescence analysis of *anti*-B[*a*]P tetrol (tetrol I-1. see abbreviations) released after acid hydrolysis of DNA samples [1]. Analysis was carried out as already described [2] with some minor modifications, mainly regarding the automation of HPLC analysis, to minimize the batch effect, and the use of a Perkin Elmer L-7485P fluorimeter to improve sensitivity (see HPLC/fluorescence analysis of anti-B[a]PDE–DNA adduct in Supplementary Material). Briefly, about 100 μg of DNA from leukocytes were dissolved in 0.1N HCl, and acid hydrolysis was carried out at 90 °C for 4 h. A Waters 717 Autosampler (Millipore Waters. Italy) coupled to a Waters Millipore automated gradient controller, a Waters 600 Multisolvent Delivery pump, and a model 510 solvent-delivery system were used. B[*a*]P-tetrol-I-1 was determined by comparison with a standard curve generated from the fluorescence areas of an authentic B[*a*]P-tetrol-I-1 standard (NCI Chemical Carcinogen Reference Standard Repository. Kansas City. MO. USA), analyzed in triplicate during analysis of each set of samples. In total, eight batches of 75 samples each were examined. The minimum correlation coefficient was 0.998 and the mean coefficient of variation (CV) for analyses repeated on different days was 10%. The highest CV value was 5.70% for those samples (*n* = 8) with more than 200 μg DNA repeated twice. In the present study, the detection threshold of B[*a*]P-tetrol-I-1 was 0.25 pg (signal/noise >3), so that this assay can measure 0.25 adducts/10^8^ nucleotides in 100 μg DNA (1 fmol/μg DNA = 30 adducts/10^8^ nucleotides), *anti*-B[*a*]PDE–DNA adduct levels. Samples with non-detectable DNA adducts were given a value of one-half the limit of detection of the method (LOD/2 = 0.125). Adduct levels were treated in the analyses both continuously and categorically (positive or non-detectable). Positive subjects were those with adduct levels ≥0.5 adducts/10^8^ nucleotides.

## Leukocytes Telomere Length (LTL)

LTL was measured by using quantitative Real-Time PCR method as previously described [3].This assay measures relative LTL in genomic DNA by determining, respectively, the ratio of telomere repeat copy number (T) to single nuclear copy gene (S), T/S ratio, in a given sample relative to a reference DNA. The single-copy gene used was human (beta) globin (hbg). As reference DNA, we used a pool DNA from 50 participants randomly selected from the study population (500 ng for each sample). A fresh standard curve, from the pool of control samples, ranging from 30 to 0.23 ng/µl (serial dilutions 1:2), was included in every “T” and “S” PCR run, against a negative control (water); 9 ng of DNA sample was added to each reaction. Each sample was run in triplicate. In brief, a high-precision MICROLAB STARlet Robot (Hamilton Life Science Robotics, Bonaduz AG, Switzerland) was used for transferring volume of 7 µl reaction mix and 3 µl DNA (3 ng/µl) in a 384-well format plate as previously described [4,5]. All PCR reactions were performed on a 7900HT Fast Real Time PCR System (Applied Biosystems). A primer pair of beta‐globin single copy gene (hbgu and hbgd) [6] and telomere primer pair (telg and telc) as those described in [7] were used in the reaction mix. The thermal cycling profile for both amplicons began with 50°C for 2 min followed by incubation at 95°C for 2 min to activate the AmpliTaq DNA polymerase. For telomere PCR, activation was followed by 2 cycles of 15 s at 95°C and 15 s at 49°C and 35 cycles of 15 s at 95°C, 10 s at 62°C, and 15 s at 74°C. For hbg, activation was followed by 35 cycles of 15 s at 95°C and 1 min at 58°C. At the end of each real-time PCR reaction, a melting curve was added for both T and S PCRs to verify the specificity of amplification. The average of the three T measurements was divided by the average of the three S measurements to calculate the average T:S ratio, i.e., relative telomere length. A measure was considered acceptable if the SD among triplicate measures was <0.25. The coefficient of variation for the average T:S ratio of samples analyzed over three consecutive days was 10%, similar to the reproducibility originally reported for this method. LTL was treated in the analyses both as categorical tl50 (higher or lower than median: 0 = below 0.896; 1 = equal/above 0.896) or continuous variable.

## Leukocyte mtDNAcn (LmtDNAcn)

LmtDNAcn was measured in the same DNA of LTL analysis using real-time quantitative PCR (qRT-PCR) as previously described [8]. This assay measures relative mtDNAcn by determining the ratio of mitochondrial (MT) copy number to single copy gene (S) copy number in experimental samples relative to the MT/S ratio of a reference pooled sample. In brief, this method is based on quantification of MT and S quantities expressed as cycle threshold (Ct; i.e., number of cycles required for the fluorescent signal to cross the threshold) derived from a standard curve obtained from serial dilutions of a reference DNA. The single-copy gene used in this study was human (beta) globin (hbg). The Mt PCR mix was: iQ SYBR Green Supermix (Bio-Rad) 1×, MtF3212 500 nmol/L, MtR3319 500 nmol/L. The S (hbg) PCR mix was: iQ SYBR Green Supermix (Bio-Rad) 1×, hbgF 500 nmol/L, hbgR 500 nmol/L, 9 ng DNA was loaded in a 10 μL PCR reaction. As a reference sample, we used the same pooled DNA from 50 participants used for telomere analysis randomly selected from this same study (500 ng for each sample) to create at every MT and S PCR run, a fresh standard curve, which ranged from 30 ng/μL to 0.23ng/μL. The primers for qRT-PCR analysis of mtDNAcn and hbg were previously described [8]. All PCRs were performed on 7900HT Fast Real-Time PCR System (Applied Biosystems). The thermal cycling conditions for mtDNAcn PCR were: 3 minutes at 98°C to activate the hot-start iTaq DNA polymerase, followed by 35 cycles comprised of 15-second denaturation at 95°C and 60-second anneal/extend at 60°C. The thermal cycling conditions for the hbg PCR were the same described above for LTL analysis 3 minutes at 98°C to activate the hot-start iTaq DNA polymerase, followed by 35 cycles comprised of 15-second denaturation at 95°C and 60-second anneal/extend at 58°C. Each run was completed by melting curve analysis to confirm the amplification specificity and absence of primer dimers. All samples were run in triplicates on 384-plate. The average of the three MT measurements was divided by the average of the three S measurements to calculate the MT/S ratio for each sample. The CV for the MT/S ratio in duplicate samples analyzed on two different days was 6%. LmtDNAcn was treated as a continuous data variable in the statistical analysis.

**References**

1. Pavanello S, Pulliero A, Saia BO, Clonfero E (2006) Determinants of anti-benzo[a]pyrene diol epoxide-DNA adduct formation in lymphomonocytes of the general population. Mutat Res 611(1-2):54-63. https://doi.org/10.1016/j.mrgentox.2006.06.034.

2. Pavanello S, Pulliero A, Siwinska E, Mielzynska D, Clonfero E (2005) Reduced nucleotide excision repair and GSTM1-null genotypes influence anti-B[a]PDE-DNA adduct levels in mononuclear white blood cells of highly PAH-exposed coke oven workers. Carcinogenesis 26(1):169-75. https://doi.org/10.1093/carcin/bgh303.

3. Pavanello S, Angelici L, Hoxha M, Cantone L, Campisi M, Tirelli AS, et al (2018) Sterol 27-Hydroxylase Polymorphism Significantly Associates With Shorter Telomere, Higher Cardiovascular and Type-2 Diabetes Risk in Obese Subjects. Front Endocrinol (Lausanne) 9:309. https://doi.org/10.3389/fendo.2018.00309.

4. Bollati V, Iodice S, Favero C, Angelici L, Albetti B, Cacace R, et al (2014) Susceptibility to particle health effects, miRNA and exosomes: rationale and study protocol of the SPHERE study. BMC Public Health 14:1137. https://doi.org/10.1186/1471-2458-14-1137.

5. Pergoli L, Cantone L, Favero C, Angelici L, Iodice S, Pinatel E, et al (2017) Extracellular vesicle-packaged miRNA release after short-term exposure to particulate matter is associated with increased coagulation. Part Fibre Toxicol 14(1):32. <https://doi.org/10.1186/s12989-017-0214-4>.

6. Pavanello S, Pesatori AC, Dioni L, Hoxha M, Bollati V, Siwinska E, et al (2010) Shorter telomere length in peripheral blood lymphocytes of workers exposed to polycyclic aromatic hydrocarbons.

Carcinogenesis 31(2):216-21. https://doi.org/10.1093/carcin/bgp278.

7. Cawthon RM (2009) Telomere length measurement by a novel monochrome multiplex quantitative PCR method. Nucleic Acids Res 37(3):e21. https://doi.org/10.1093/nar/gkn1027.

8. Pavanello S, Dioni L, Hoxha M, Fedeli U, Mielzynska-Svach D, Baccarelli AA (2013) Mitochondrial DNA copy number and exposure to polycyclic aromatic hydrocarbons. Cancer Epidemiol Biomarkers Prev 22(10):1722-9. doi: 10.1158/1055-9965.EPI-13-0118.

**Supplementary Tables**

| Table 1S. Spearman's rank coefficients and significance level for pairwise correlation of the different environmental exposures to PAHs (see Methods for definitions) as well as age and sex. | | | | | | |
| --- | --- | --- | --- | --- | --- | --- |
|  | Diet | Indoor | Traffic | Home | Outdoor | Age |
| Indoor | 0.18  **<0.001** |  |  |  |  |  |
| Traffic | –0.11  **0.008** | –0.09  **0.037** |  |  |  |  |
| Home | –0.13  **0.003** | –0.28  **<0.001** | 0.25  **<0.001** |  |  |  |
| Outdoor | 0.07  0.088 | 0.11  **0.013** | –0.01  0.824 | 0.03  0.498 |  |  |
| Age | –0.24 **<0.001** | –0.05  0.233 | 0.13  **0.003** | 0.14  **0.002** | 0.03  0.451 |  |
| Sex | 0.09  **0.035** | 0.08  0.060 | –0.06 0.202 | 0.01  0.864 | 0.35  **<0.001** | 0.23  **<0.001** |

| Table 2S. Four groups of SEM results (structural equations, measurement, variances and covariances) for the analysis of tl50; standardized beta coefficients (with “minus” sign indicating inverse relationship) with lower and upper limit of 95% confidence intervals (95%CI) and p-values. SEM’s goodness-of-fit statistics at bottom of table. | | | | | | |
| --- | --- | --- | --- | --- | --- | --- |
|  | Endogenous variables | Exogenous variables | Beta Coef. | 95%CI | | p-value |
|  |  |  |  | Lower | Upper |  |
| Structural Equations | anti-B[a]PDE–DNA | PAH | 0.178 | 0.005 | 0.055 | 0.005 |
|  |  | smoking_gp | 0.149 | 0.000 | 0.066 | 0.0001 |
|  |  | gstm1 | –0.098 | –0.021 | –0.182 | 0.021 |
|  |  | sex | –0.087 | 0.057 | –0.177 | 0.057 |
|  | T/S_50 percent_ | anti-B[a]PDE–DNA | –0.092 | –0.175 | –0.010 | 0.028 |
|  |  | smoking_gp | 0.187 | 0.105 | 0.268 | 0.0001 |
|  |  | age | –0.135 | –0.218 | –0.053 | 0.001 |
|  |  | sex | –0.117 | –0.201 | –0.033 | 0.006 |
| Measurement | Diet ← PAH | | 0.439 | 0.317 | 0.561 | 0.0001 |
|  | Indoor ← PAH | | 0.436 | 0.311 | 0.561 | 0.0001 |
|  | Home ← PAH | | –0.433 | –0.573 | –0.292 | 0.0001 |
|  | Traffic ← PAH | | –0.309 | –0.429 | –0.189 | 0.0001 |
|  | Outdoor ← PAH | | 0.212 | 0.071 | 0.354 | 0.003 |
| Errors | ε1 = var(e.diet) | | 0.807 | 0.707 | 0.921 |  |
|  | ε2 = var(e.indoor) | | 0.810 | 0.708 | 0.927 |  |
|  | ε3 = var(e.home) | | 0.813 | 0.700 | 0.944 |  |
|  | ε4 = var(e.traffic) | | 0.904 | 0.833 | 0.982 |  |
|  | ε5 = var(e.outdoor) | | 0.955 | 0.897 | 1.017 |  |
|  | ε6=var(e.anti-B[a]PDE–DNA) | | 0.931 | 0.882 | 0.983 |  |
|  | ε7 = var(e.T/S) | | 0.932 | 0.893 | 0.973 |  |
|  | ε8 = var(PAH) | | 1 | . | . |  |
| Covariances | cov(sex. PAH) | | 0.230 | 0.082 | 0.378 | 0.002 |
|  | cov(age. PAH) | | –0.318 | –0.439 | –0.197 | 0.0001 |

Goodness of fit statistics:

Likelihood ratio (LR) test of model vs. saturated: chisquare(28) = 139.400 (p <0.0001)

Standardized root mean squared residual (SRMR) = 0.055

Coefficient of determination (CD) = 0.524

| Table 3S. Four groups of SEM results (structural equations, measurement, variances and covariances) for analysis of LmtDNAcn: standardized beta coefficients (with “minus” sign indicating inverse relationship) with lower and upper limit of 95% confidence intervals (95%CI) and p-values. SEM’s goodness-of-fit statistics at bottom of table. | | | | | | |
| --- | --- | --- | --- | --- | --- | --- |
|  | Endogenous variables | Exogenous variables | Beta Coef. | 95%CI | | p-value |
|  |  |  |  | Lower | Upper |  |
| Structural Equations | anti-B[a]PDE–DNA | sex | –0.087 | –0.177 | 0.002 | 0.056 |
|  |  | smoking | 0.150 | 0.067 | 0.233 | 0.0001 |
|  |  | gstm1 | –0.093 | –0.176 | –0.009 | 0.030 |
|  |  | PAH | 0.209 | 0.083 | 0.336 | 0.001 |
|  | Mt_­­DNA | anti-B[a]PDE–DNA | –0.100 | –0.183 | –0.017 | 0.018 |
|  |  | Sex | –0.146 | –0.228 | –0.064 | 0.0001 |
| Measurement | Diet <- PAH | | 0.334 | 0.216 | 0.452 | 0.0001 |
|  | Indoor <- PAH | | 0.547 | 0.417 | 0.676 | 0.0001 |
|  | Home <- PAH | | –0.469 | –0.609 | –0.329 | 0.0001 |
|  | Traffic <- PAH | | –0.281 | –0.409 | –0.152 | 0.0001 |
|  | Outdoor <- PAH | | 0.187 | 0.045 | 0.329 | 0.010 |
| Variances | ε1 = var(e.diet) | | 0.889 | 0.813 | 0.971 |  |
|  | ε2 = var(e.indoor) | | 0.701 | 0.573 | 0.858 |  |
|  | ε3 = var(e.home) | | 0.780 | 0.659 | 0.923 |  |
|  | ε4 = var(e.traffic) | | 0.921 | 0.852 | 0.996 |  |
|  | ε5 = var(e.outdoor) | | 0.965 | 0.914 | 1.019 |  |
|  | ε6 = var(e. anti-B[a]PDE–DNA) | | 0.919 | 0.863 | 0.979 |  |
|  | ε7 = var(e. LmtDNAcn) | | 0.969 | 0.941 | 0.998 |  |
|  | ε8 = var(PAH) | | 1 | . | . |  |
| Covariances | cov(sex. PAH) | | 0.194 | 0.052 | 0.335 | 0.007 |

Goodness of fit statistics:

Likelihood ratio test of model vs. saturated: chi2(28) = 116.2, p <0.0001.

Standardized root mean squared residual (SRMR) = 0.055

Coefficient of determination (CD) = 0.529
